# Biochemical Analysis of the NAD^+^-Dependent Malate Dehydrogenase, a Substrate of Several Serine/Threonine Protein Kinases of *Mycobacterium tuberculosis*

**DOI:** 10.1371/journal.pone.0123327

**Published:** 2015-04-10

**Authors:** Xiao Ming Wang, Karine Soetaert, Priska Peirs, Michaël Kalai, Véronique Fontaine, Jean Paul Dehaye, Philippe Lefèvre

**Affiliations:** 1 Scientific Institute of Public Health, Direction of Communicable and Infectious Diseases, Rue Engeland 642, 1180 Brussels, Belgium; 2 Unité de Microbiologie Pharmaceutique et Hygiène, Faculty of Pharmacy, Université Libre de Bruxelles, Boulevard du Triomphe, CP205/2, 1050 Brussels, Belgium; Bose Institute, INDIA

## Abstract

PknD is one of the eleven eukaryotic-like serine/threonine protein kinases (STPKs) of *Mycobacterium tuberculosis (Mtb)*. *In vitro* phosphorylation assays with the active recombinant PknD showed that the intracellular protein NAD^+^-dependent malate dehydrogenase (MDH) is a substrate of this kinase. MDH, an energy-supplying enzyme, catalyzes the interconversion of malate and oxaloacetate and plays crucial roles in several metabolic pathways including the citric acid cycle. The phosphorylation site was identified on threonine residues and the phosphorylation inhibited the MDH activity. *In vitro*, the recombinant MDH could also be phosphorylated by at least five other STPKs, PknA, PknE, PknH, PknJ, and PknG. Immunoprecipitation analysis revealed that MDH was hyperphosphorylated in the bacteria at the beginning of the stationary and under oxygen-limited conditions by STPKs other than PknD. On the contrary, when PknD-deficient mutant mycobacteria were grown in a phosphate-depleted medium, MDH was not detectably phosphorylated. These results suggest that although the MDH is a substrate of several mycobacterial STPKs, the activity of these kinases can depend on the environment, as we identified PknD as a key element in the MDH phosphorylation assay under phosphate-poor conditions.

## Introduction

*Mycobacterium tuberculosis (Mtb)*, a causative agent of tuberculosis, is responsible for considerable worldwide human morbidity and mortality. Nearly a third of the world population is infected with persistent or latent *Mtb*, referred to as latent tuberculosis. Reactivation of latent infection is the major source of active tuberculosis in adults [1]. One of the main obstacles in the global control of tuberculosis is linked to the emergence of multi-drug resistant strains and the therapeutic failure of persistent infections treatment using conventional anti-tuberculosis drugs. During infection, *Mtb* resides in the hypoxic necrotic core of complex immunological structures, called granulomas, either within macrophages or in the extracellular compartment [2].

The mechanisms involved in the development of drug-tolerant bacteria are still not clear. They are thought to arise from persistent or latent bacteria with slower replication and metabolic rates. Environment induced mechanisms, such as those in response to stress (hypoxia) could lead to the development of a subpopulation of stress-tolerant cells, able to persist in a wide range of unfavorable conditions, including those where antibiotics are present [3]. It is therefore believed that the regulation of a flexible metabolism in response to environmental changes plays a significant contribution to the virulence of *Mtb* [4]. The mechanisms behind this metabolic plasticity are mostly unknown [5]. The persistent bacteria use primarily fatty acids as their carbon source [6], with the catabolism of the fatty acids releasing acetyl-CoA. The total oxidation of the acetyl units through the tricarboxylic acid (TCA) cycle provides the bacteria with the major source of energy in aerobic pathways. Upon oxygen limitation, *Mtb* accumulates triacylglycerols (TAG) [7] and the intracellular ATP level decreases [8]. Bacterial growth arrest during mouse lung infection or nutrient starvation has been shown to be associated with increased expression and activity not only of enzymes involved in TAG synthesis, but also of isocitrate lyase and phosphoenolpyruvate carboxykinase. Concomitant with the latter, down-regulation of most TCA cycle proteins [8, 9] takes place, emphasizing the impact of anaplerotic reactions and of the glyoxylate shunt that fixes carbon into biomass [10].

The two-component systems represent the classical prokaryotic mechanism for detection and response to environmental changes. Serine/threonine and tyrosine protein kinases associated with their phosphatases are also important regulatory systems in bacteria [11–13]. *Mtb* contains eleven serine/threonine protein kinases (STPKs) [12], named PknA to PknL. Two of them are soluble proteins, PknG and PknK, the nine other proteins being transmembrane kinases. Recent studies suggested extensive phosphorylation with overlapping specificity by *Mtb* STPKs [14] and reported the identification and characterization of the phosphorylation sites in substrates related to various metabolic pathways in mycobacteria [12, 14]. These include MmpL7, a transporter of polyketide virulence factors such as phthiocerol dimycocerosate [15], the anti-anti-sigma factor Rv0516c [16], the alternate SigH and SigF sigma factors, which are key regulators of the stress response [17, 18] and the transcriptional regulator VirS known to regulate the expression of the mycobacterial monooxygenase (*mymA*) operon [19]. Recently, PknA was shown to regulate the cell division via Wag31 [20]. PknE is involved in the nitric oxide stress response of *Mtb* in macrophage [21] and PknG contributes to mycobacterial survival within macrophages by preventing phagosome-lysosome fusion [22]. Noteworthy, STPK autophosphorylation not only activates the kinase domains but also creates binding sites for substrate proteins containing FHA domains, consisting in phosphothreonine-peptide recognition motifs [23–26]. Five genes coding for FHA containing proteins have been located within the *Mtb* genome, including Rv1827 encoding GarA, a *Mtb* TCA regulator [27] which is itself phosphorylated by STPKs [28], and Rv0020c encoding a protein of unknown function [29].

A further characterization of substrates of the various STPKs is critical to understand the mechanisms by which STPK-dependent phosphorylation might regulate the metabolic activity of mycobacteria, especially during the shift from aerobic to anaerobic conditions. In the present study, we focused on the *Mtb* PknD and its substrates. We showed that the NAD^+^-dependent malate dehydrogenase (MDH) was phosphorylated by several kinases including PknD. The MDH activity was reduced by the phosphorylation of the enzyme on threonine residue(s) and the phosphorylated MDH bound to the FHA containing proteins, GarA and Rv0020c. Finally, we studied the impact of environmental growth conditions on the phosphorylation of the MDH. Using a PknD deficient mutant *Mtb* strain, we identified that PknD played a specific role under poor phosphate culture conditions.

## Material and Methods

### Ethics statement

The animal care and ethic committee of the “Institut Pasteur de Bruxelles” approved this study (permit number: LA1230177).

### Bacterial strains, media and chemicals

The *E*. *coli* DH5α strain was used to propagate plasmids in cloning experiments, and the *E*. *coli* BL21 (DE3) was used to overproduce the recombinant (GST-tag) serine/threonine protein kinases, Rv1827, Rv0020c and MDH. All strains were grown and maintained in Luria-Bertani medium supplemented with 100 μg/ml ampicillin at 37°C. All restriction enzymes, T4 DNA ligase, Klenow fragment and Pfu polymerase were from Promega (Leiden, The Netherlands). Plasmids were purified using the QIAprep Spin Miniprep kit (Qiagen, Venlo, The Netherlands). Primers were purchased from Genset (Paris, France), [γ-^33^P]ATP and [γ -^32^P]ATP from Amersham Biotech (Diegem, Belgium).

*Mtb* H37Rv wild-type (WT) and the *Mtb* PknD-deficient (KO) strain [30] were cultured in Middlebrook 7H9 medium (Difco, BD Biosciences, Erembodegem, Belgium) supplemented with 10% Middlebrook oleic acid-albumin-dextrose-catalase (OADC) enrichment (Difco, BD Biosciences) and 0.05% Tween 80. The mutant mycobacteria were cultured in 20 μg/ml kanamycin. For the phosphate deprivation cultures, bacteria were first grown in Middlebrook 7H9 broth to an optical density at 600 nm (OD_600 nm_) of 0.9, washed three times with a buffer (50 mM Tris, 15 mM KCl, 10 mM (NH_4_)_2_SO_4_, 1 mM MgSO_4_ and 0.05% Tween 80, pH 7.2), resuspended in the modified Sauton medium lacking potassium dihydrogen phosphate and further incubated for 3h at 37°C. Oxygen-limited Wayne cultures were grown in Dubos Tween-albumin broth (Difco, BD Biosciences) in sealed test tubes (20 mm by 125 mm) containing a magnetic stirring bar and a head-space air ratio of 0.5 to that of medium. The tubes were placed on a magnetic stirring plate and the bars spun at a speed sufficient to keep the bacilli suspended uniformly throughout the medium without perturbing the liquid surface, as previously described [31,32]. A sterile solution of methylene blue was added to a final concentration of 1.5 μg/ml. Reduction and discoloration of this dye served as a visual indicator for oxygen depletion. *M*. *bovis* BCG 1173P2 (Pasteur Institute, Paris, France) was grown as a pellicle at 37°C on Sauton medium.

### Chromatographies

*M*. *bovis* BCG 1173P2 cell extracts were prepared from 14-days-old cultures. The bacterial wet pellets (300 g) were suspended in 20 mM phosphate buffer, 450 mM NaCl, pH 7.3, homogenized with a potter homogenizer, then disrupted by pressure at 83–110 Mpa in a French press and clarified by centrifugation at 4,000 g. After a second centrifugation at 100,000 g for 90 min, resulting supernatants were used for hydrophobic and ion-exchange chromatographies. The soluble cell extract was applied to a phenyl-Sepharose column Cl-4B (10 x 30 cm, GE Healthcare, Diegem, Belgium), and unbound materials were recovered by washing the gel with 20 mM phosphate, 450 mM NaCl buffer, pH 7.3. A gel-filtration chromatography using a desalting column G25 (K26/10, GE Healthcare, Diegem, Belgium) replaced the phosphate buffer by 20 mM Tris pH 7.3. This solution was then loaded on a Resource-Q column (1 ml, GE Healthcare, Diegem, Belgium) and elution was performed using a 0–0.5 M NaCl gradient. The absorbance was monitored at 280 nm. Proteins separated by the anion-exchange chromatography into different fractions were then subjected to an *in vitro* phosphorylation assay.

### Construction and expression of His_6_-tagged MDH, Flag-Rv1827 and Flag-Rv0020c expression plasmids

The *mdh*, *Rv1827* and *Rv0020c* genes were synthesized by PCR amplification using *M*. *tuberculosis* H37Rv genomic DNA as a template. These DNA fragments were digested with restriction enzymes and ligated in frame into vector pRSETc and pFLAG, generating pRSETc-*mdh*, pFLAG*-Rv1827* and pFLAG*-Rv0020c*. The sequences were verified by DNA sequencing. The *E*. *coli* BL21(DE3) cells were transformed with the pRSETc-*mdh*, pFLAG*-Rv1827* and pFLAG-*Rv0020c* vectors expressing respectively His_6_-MDH, Flag-Rv1827 and Flag-Rv0020c fusion proteins. These *E*. *coli* strains were grown in 200 ml at 37°C with shaking until OD_600nm_ reached 0.6–0.8. One mM (final concentration) IPTG was then added and growth was further continued for two additional hours.

### Purification of His_6_-MDH

Cells were harvested by centrifugation at 6,000 g for 15 min and washed twice with 20 ml Tris buffer (20 mM Tris-HCl, pH 7.5). The cell pellet was resuspended in 20 ml saline Tris buffer (20 mM Tris-HCl pH 7.5, 500 mM NaCl) and disrupted by sonication for 5 min. After centrifugation at 4°C for 30 min at 12,000 g, the supernatant containing the fusion proteins was loaded onto a 1 ml HiTrap chelating HP column (GE Healthcare, Diegem Belgium) previously saturated with 100 mM NiSO_4_. The His_6_-MDH elution was carried out in the saline Tris buffer with a 0–0.5 M imidazole gradient. Eluted fractions were analyzed by SDS-PAGE using 12% polyacrylamide gels. Pure fractions were pooled and dialyzed against a 20 mM Tris-HCl, pH 7.0, 200 mM NaCl, 1% glycerol buffer. Protein concentrations were determined by the Bradford method using bovine serum albumin as standard.

### Antibody preparation and Western blot analyses

Two rabbits were injected with 0.2 ml purified MDH (1 mg/ml in PBS buffer). The injection was repeated after 4 and 7 weeks. After 10 weeks, 10 ml blood were drawn from the rabbits and stored overnight at 4°C. The clotted blood was centrifuged at 3,000 g for 10 min and the serum was collected.

Western blot analyses were carried out with different antibodies: a mAb anti-Xpress against His_6_-tag epitope (Invitrogen SA, Merelbeke, Belgium), a mAb anti-Flag M2 (Sigma-Aldrich BVBA, Diegem, Belgium), an anti-phosphothreonine antibody (Calbiochem, Overijse, Belgium) and anti-MDH polyclonal antibodies.

### Detection of the MDH activity after non-denaturing PAGE

Proteins were separated on 8% polyacrylamide gels under non-denaturing conditions (absence of SDS from all buffers, samples were not boiled and ß-mercaptoethanol was absent from the sample buffer). The MDH activity was detected by incubating the gel at 30°C in the dark in 15 ml of 100 mM Tris/acetate, pH 8.0 containing 25 mM L-malate, 0.22 mM NAD, 0.005% phenazine methosulfate and 0.008% nitro blue tetrazolium, as previously described by Cannata and Cazzulo [33].

### *In vitro* phosphorylation assay

One μg protein was incubated at 37°C for 30 min with 250 ng of recombinant GST-PknD in the presence of 5 μCi [γ-^33^P]ATP in a 25 mM Hepes, 60 mM KCl, 1 mM DTT, 2.5 mM Mn acetate (pH 7.5) buffer. The products of the reactions were then separated by SDS-PAGE (12% polyacrylamide gels). The gels were dried and exposed on X-ray film (Kodak XAR) for autoradiography.

### Analysis of the N-terminal amino acids sequence

Proteins were transferred from the SDS-PAGE gels to polyvinylidene difluoride (PVDF) membranes using a LKB Multiphor II electrophoresis unit by semidry electroblotting. N-terminal amino acids sequence analysis was performed by Edman degradation in an automated 476-A pulsed liquid sequencer equipped with an on-line PTH-amino acid analyzer (Applied Biosystems, Foster City, CA, USA).

### Identification of the phosphorylated amino acid in MDH

The ^32^P-phosphorylated protein samples were separated by SDS-PAGE and transferred to a polyvinylidene difluoride (PVDF) membrane. Phosphorylated proteins bound to the membrane were detected by autoradiography. The ^32^P-labeled proteins bands were excised from the PVDF blot and hydrolyzed in 6 M HCl for 24 h at 110°C. The acid-stable phospho-amino acids were separated by two-dimensional electrophoresis with the first dimension at pH 1.9 (750 V.h) in 7.8% acetic acid, 2.5% formic acid, followed by the second dimension at pH 3.5 (500V.h) in 5% acetic acid, 0.5% pyridine. After migration, radioactive molecules were detected by autoradiography. Authentic phosphoserine, phosphothreonine and phosphotyrosine were run in parallel and visualized using ninhydrin staining.

### Dot-blot immunoanalysis

Zero or one μg of the His_6_-MDH non-phosphorylated or phosphorylated by 0.25 μg of PknD were spotted on the nitrocellulose membranes. The membranes were then incubated with 1 μg/ml of Flag-Rv1827 or Flag-Rv0020c for 16 h at 4°C with agitation. After washing, the Flag epitope was used to detect the bound Flag fusion proteins by chemiluminescence immunoanalysis using a mAb anti-Flag M2 (Sigma-Aldrich BVBA, Diegem, Belgium).

### MDH activity

Malate dehydrogenase (EC 1.1.1.37) activity was assayed by spectrophotometry at 340 nm using an Uvikon 930 spectrophotometer. The assay was carried out in a 1 ml cuvette at room temperature in 100 mM glycine pH 9.0. The MDH activity was examined by measurement of the NADH consumption/production rate accompanying the reduction/oxidation of oxaloacetate/malate. An extinction coefficient of 6.2 x 10^3^ M^-1^cm^-1^ was used to determine all kinetic parameters. To test the effect of phosphorylation on the activity of MDH, 0.15 μg of the MDH phosphorylated by 30 ng of PknA, PknD, PknE, or PknH was used for measurement in a 1 ml-cuvette containing 0.14 mM NADH and 0.8 mM oxaloacetate. A negative control was also run in parallel with the autophosphorylated kinase alone, in the absence of MDH.

### Immunoprecipitation

*Mtb* cell pellets were resuspended in 50 mM Tris-HCl pH 7.4, 200 mM NaCl, 5 mM EDTA, in the presence of the Complete protease inhibitor cocktail and phosphatase inhibitors (Roche Applied Science, Mannheim, Germany). Cells were lyzed by sonication and membranes debris were eliminated by centrifugation at 13,000 rpm for 10 min. The soluble extracts were incubated at 4°C during 4 h with protein-A sepharose beads pre-coupled with polyclonal antibodies against the MDH. After washing the beads, the antibody/antigen complexes were recovered by boiling for 5 min before SDS-PAGE electrophoresis.

## Results

### Identification of the *M*. *bovis* BCG PknD substrates by *in vitro* phosphorylation

**Fig 1:**
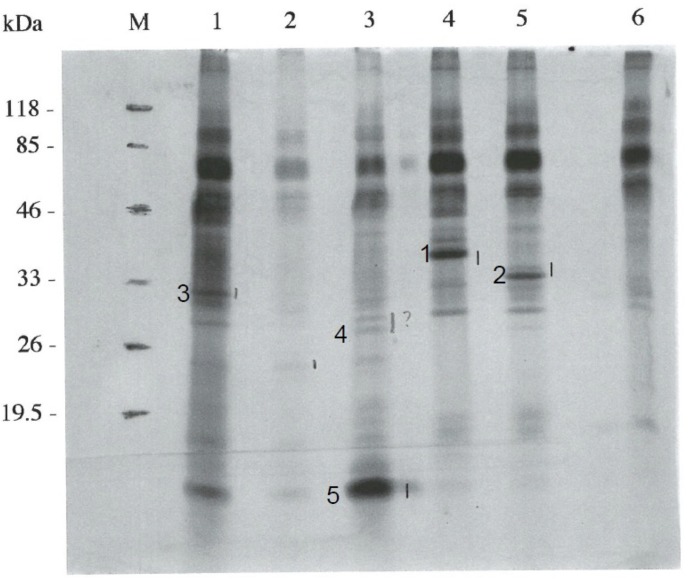
Phosphorylation of mycobacterial proteins by PknD. After separation by chromatography of the proteins from a homogenate from *M*. *bovis* BCG 1173P2, some fractions were pooled and incubated in the presence of (γ-^33^P)- ATP and of PKnD. Proteins were separated by SDS-PAGE and the phosphoproteins were visualized by autoradiography. Lanes M : standards of various molecular weights ; 1 : chromatographic fractions 42–44; 2 : chromatographic fractions 45–47; 3 : chromatographic fractions 48–50; 4: chromatographic fractions 66–69; 5 : chromatographic fractions 71–75; 6: no fraction from the chromatography. After transfer to a PVDF membrane, the bands 1 to 5 were sequenced using the Edman degradation method.

To investigate the substrates of the PknD protein kinase, a soluble extract of *M*. *bovis* BCG was applied to a phenyl-Sepharose column CL-4B and the unbound pass-through material was separated by anion-exchange chromatography on a Resource-Q column. Samples from eluted fractions were tested in an *in vitro* phosphorylation assay using recombinant PknD protein and [**γ**-^32^P]ATP. As shown in Fig 1, several proteins in the various chromatographic fractions were phosphorylated by PknD. Five of these proteins were identified after sequencing using Edman degradation (Table 1). A phosphorylated protein present in lane 4 (chromatographic fractions 66–69, band 1) with an apparent MW around 37 kDa seemed to be the best substrate of the kinase. Its N-terminal sequence (SASPLKVAVT) was used to search by similarity with the BLAST (NCBI) program in the Sanger *Mtb* H37Rv database for the identity of the protein. The peptide fully matched the deduced N-terminal amino acid sequence of the Rv1240 ORF encoding the NAD^+^-dependent malate dehydrogenase (MDH). Further studies were thus performed on this enzyme, especially on its regulation by PknD.

**Table 1:** Determination of the major proteins phosphorylated by PknD in a homogenate from *M*. *bovis*.

Band	Sequence	ID	Database results	N° AA in the sequence	Site phosphorylation
1	SASPLKVAVT	Rv1240	Malate dehydrogenase	2–11	6 Ser, 3 Thr
2	AEVLVLVEHAEG	Rv3038C	Electron transfer flavoprotein alpha subunit	2–13	5 Ser, 6 Thr
3	TLNLXVDEVLT(T)	Rv3368C	Hypothetical protein Nitroreductase family	2–13	4 Ser, 3 Thr
4	AEYTLPDLDWDY	Rv3846	Superoxide dismutase chain A/B	2–13	2 Thr
5	ALPQLTDEQRAAALE	Rv1388	MihF: mycobacterial integration host factor	87–101	2 Ser, 2 Thr

Various fractions of the chromatography of a homogenate from *M*. *bovis* were incubated with PknD and the phosphorylated proteins were separated by SDS-PAGE. Five bands (numbered on the gels) were extracted and sequenced using the Edman degradation. They were identified by comparison with the genome of the H37Rv strain.

### *In vitro* phosphorylation of the recombinant MDH by PknD on threonine residue

**Fig 2:**
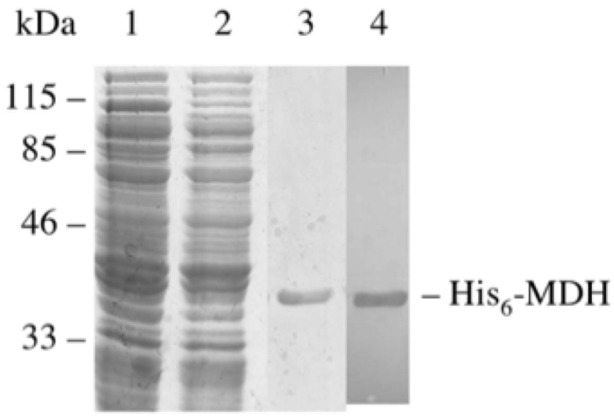
SDS-PAGE of the His_6_-MDH stained by Coomassie-Blue R-250. His_6_-MDH was overproduced as a soluble protein and purified on a Ni^2+^-immobilized HiTrap column. Lanes 1 (30 μg) and 2 (30 μg) show *E*. *coli* soluble extract before and after its loading on the HiTrap column, respectively. Lane 3 (1.5 μg) shows the purified His_6_-MDH. The purified His_6_-MDH was also observed by Western blot using a mAb anti-Xpress against His_6_-tag epitope (lane 4).

**Fig 3:**
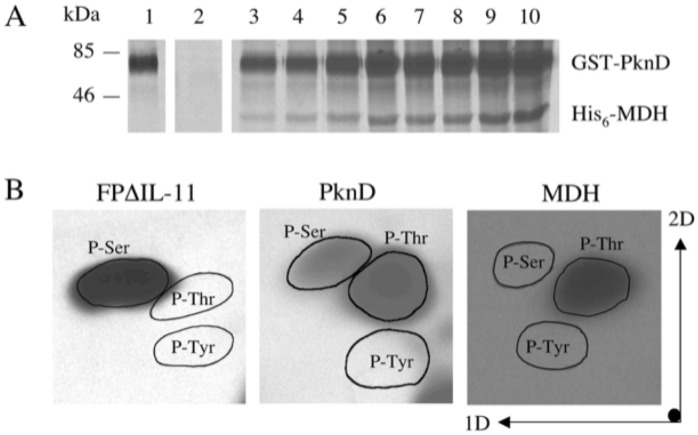
*In vitro* phosphorylation of the MDH by the GST-PknD. A: Incubation with [γ-^33^P]ATP of the PknD alone (lane 1), the MDH alone (lane 2), the MDH with the GST-PknD for 30 sec (lane 3), 1 min (lane 4), 2 min (lane 5), 4 min (lane 6), 6 min (lane 7), 8 min (lane 8), 10 min (lane 9) and 15 min (lane 10). Proteins were separated by SDS-PAGE and radioactive bands were revealed by autoradiography. B: Phosphoamino acid content of the His_6_-MDH. The MDH was labeled with [γ -^32^P]ATP, analyzed by SDS-PAGE, transferred onto a PVDF membrane, excised, hydrolyzed in acid and separated by 2D electrophoresis on a TLC plate. Radioactive molecules were detected by autoradiography, whereas phosphoserine (P-Ser), phosphothreonine (P-Thr), and phosphotyrosine (P-Tyr) as standard controls were run in parallel and visualized by ninhydrin staining. Phosphorylated FPΔIL-11 was used as a phosphoserine positive control and phosphorylated PknD as phosphoserine and phosphothreonine control.

The *Mtb* Rv1240 gene was cloned in frame into the expression vector pRSETc to produce an N-terminal His_6_-tagged recombinant protein. The protein expressed in a soluble form was purified to homogeneity with the predicted molecular mass of approximately 37 kDa (Fig 2). As shown in Fig 3A, the MDH was phosphorylated by PknD, but could not be autophosphorylated, confirming that MDH possessed no autokinase activity and that the observed phosphorylation of MDH was indeed mediated by PknD. Kinetic analysis of the phosphorylation showed that the phosphorylation occurred rapidly, reaching about 50% of its maximal rate within 4 min. After acid hydrolysis of the phosphorylated protein, the free amino acids were analyzed by two-dimensional electrophoresis. Comparison with phosphorylated standards showed that the phosphorylation had occurred on threonine residues (Fig 3B).

### The MDH could be phosphorylated *in vitro* by other *Mtb* Ser/Thr protein kinases

**Fig 4:**
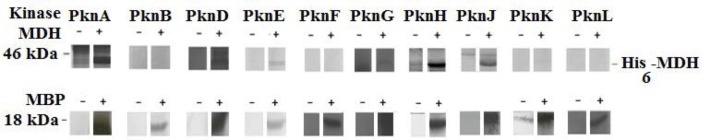
*In vitro* phosphorylation of the MDH by other *M*. *tuberculosis* STPKs. Fifty nM of PknA, PknB, PknE, PknF, PknG, PknH, PknJ, PknK or PknL were incubated with [γ -^33^P]ATP in the absence or in the presence of MDH or not, respectively, for 30 min at 37°C. After phosphorylation, proteins were separated by SDS-PAGE, and radioactive bands were revealed by autoradiography.

Protein kinases exert their influence by recognizing a specific set of protein substrates. *Mtb* consists in the largest set of phosphorylated serines, threonines and tyrosines in bacteria (over 300 phosphoproteins) and the 11 *Mtb* STPKs have often been reported to phosphorylate common substrates [13, 14]. In order to find out the specificity of the MDH phosphorylation, nine other *Mtb* STPKs (PknA, PknB, PknE, PknF, PknG, PknH, PknJ, PknK and PknL) were used for *in vitro* phosphorylation assay. The full length of these kinases were all expressed in *E*. *coli* [22, 34] and fused at the N-terminus with a GST peptide. Their kinase activities were confirmed using the myelin basic protein (MBP) as a positive control (Fig 4). As shown in Fig 4, PknA, PknD, PknE, PknG, PknH and PknJ phosphorylated the (His)_6_-MDH.

### PknD phosphorylated functional dimeric *Mtb* MDH

**Fig 5:**
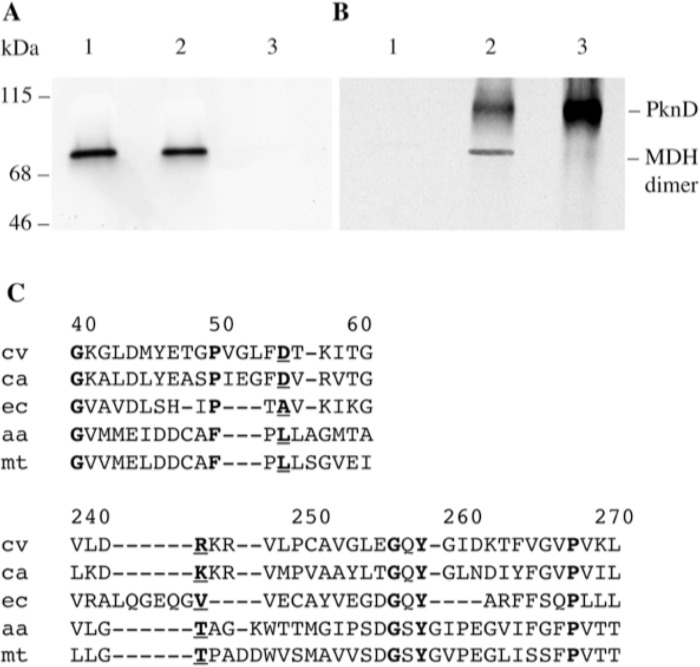
The active form of the *Mtb* MDH consists in a dimer. A: Native polyacrylamide gel (8%) was stained for MDH activity as described under Materials and Methods. B: Autoradiography of the gel. Lane 1: 2 μg of non-phosphorylated MDH; Lane 2: 2 μg of MDH phosphorylated by 0.25 μg of PknD; Lane 3: 0.25 μg of autophosphorylated PknD.

In most cases, eukaryotic MDHs are dimeric [35]. However, in some prokaryotes, the MDH can be tetrameric in its native state [36]. The functional relevance of the two MDH forms is not clear but the dimeric structure of the *E*. *coli* MDH is critical for its enzyme activity [37]. The active form of the *Mtb* MDH was a dimer as revealed by a native gel electrophoresis stained through a reaction catalyzed by the MDH activity (Fig 5A). This result suggested that the fusion of the His_6_-tag at the N-terminus of the MDH did not inhibit the formation of a dimer by MDH. As shown in Fig 5B, PknD phosphorylated the dimeric MDH *in vitro*.

### Regulation of the MDH activity by PknD

**Fig 6:**
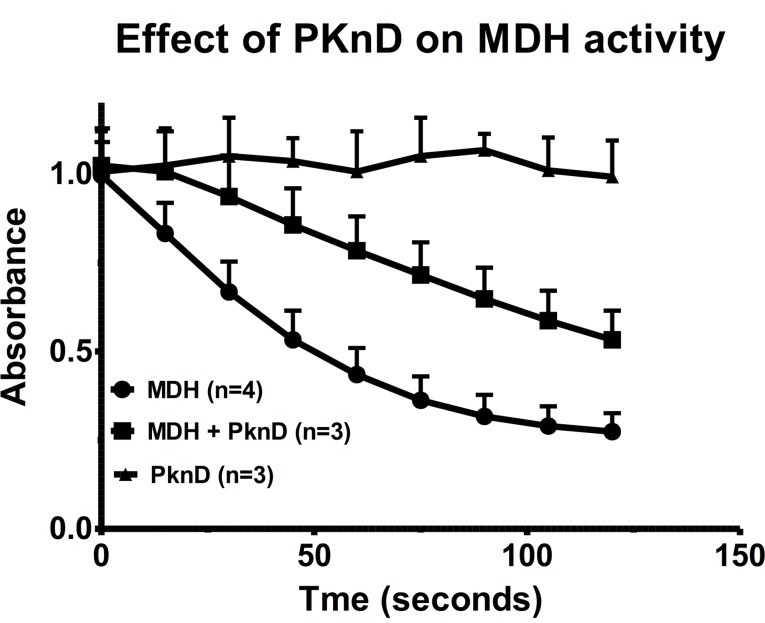
Inhibition of the *in vitro* MDH activity by phosphorylation. The MDH activity was assayed by measuring the decrease in NADH absorbance at 340 nm. The assay was carried out before (■) or after (●) the phosphorylation of MDH by the PknD at a room temperature (20°C), or in the absence of MDH but in the presence of PknD (▲). Results are the means ± s.e.m. of n experiments performed in triplicates.

The activity of the purified MDH enzyme was assayed using either oxaloacetate or malate as substrates. Table 2 summarized the values of the steady-state parameters. The *k*_cat_/*K*_m_ values for oxaloacetate and malate were about 17.40 and 0.29 respectively. The *in vitro* conditions to assay the activity of recombinant MDH favored the reduction of oxaloacetate coupled to the oxidation of NADH. After phosphorylation by PknD, the velocity of the NADH oxidation measured between 15 and 60 seconds decreased from—0.509 ± 0.025/min to—0.297 ± 0.058/min (n = 3; P < 0.01). The presence of the autophosphorylated PknD had no effect by itself on the oxidation of NADH (Fig 6). This indicated that the MDH phosphorylation by the PknD inhibited its activity (40% inhibition). In 2 other experiments, the activity of MDH was also inhibited after phosphorylation by 3 other kinases (42 and 53% inhibition for PknF; 23 and 32% inhibition for PknH; 27 and 38% inhibition for PknA). These results confirmed that the phosphorylation of MDH by STPKs regulated (inhibited) its activity.

**Table 2:** Kinetic parameters of the purified recombinant *Mtb* MDH.

Substrate/cofactor	***K***_*m*_ (μM)	***k***_*cat*_ (sec^-1^)	***k***_*cat*_ ***/ K***_*m*_ ***(***μM^-1^ sec^-1^)
Malate	1040	302	0.29
NAD^+^	130	320	2.46
NADH	80	3330	41.60
Oxaloacetate	140	2437	17.40

### The PknD phosphorylated MDH can bind to Rv1827 and Rv0020c, two proteins containing a FHA domain

**Fig 7:**
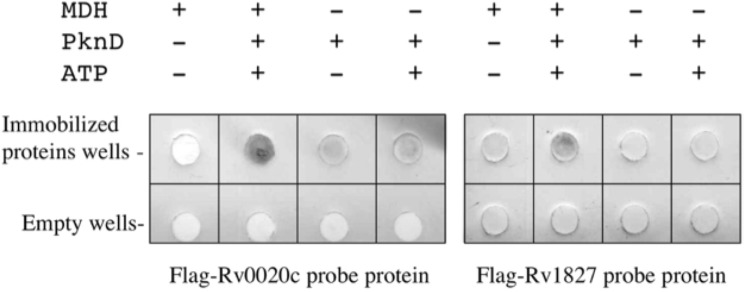
Interaction between phosphorylated MDH and two FHA-containing proteins. Zero or one μg of the non-phosphorylated or phosphorylated MDH (by PknD) was spotted on nitrocellulose membrane and further incubated with Flag-Rv1827 or Flag-Rv0020c. After washing, the Flag epitope was detected by chemiluminescent immunoanalysis using a mAb anti-Flag M2.

The FHA domain recognizes phosphothreonine on proteins [25]. Five *Mtb* proteins have been found to contain at least one FHA domain [38]. Two of the corresponding genes *Rv1827* and *Rv0020c*, have been cloned in frame with the flag epitope sequence in order to produce tagged proteins. The binding between these proteins and the His_6_-MDH was evaluated by dot-blot analysis. Phosphorylated and non-phosphorylated His_6_-MDH and controls were spotted on nitrocellulose membranes, which were then incubated with either flag-Rv1827 or flag-Rv0020c proteins. After washing, bound flag-Rv1827 and flag-Rv0020c were detected using an anti-flag monoclonal antibody. As shown in Fig 7, only the phosphorylated His_6_-MDH could bind to the Rv1827 and Rv0020c proteins. This result suggested that the association between His_6_-MDH and Rv1827 or Rv0020c was not secondary to non-specific interactions but involved the phosphothreonine residue(s) on the phosphorylated MDH.

### Phosphorylation of the *Mtb* MDH during bacterial culture

**Fig 8:**
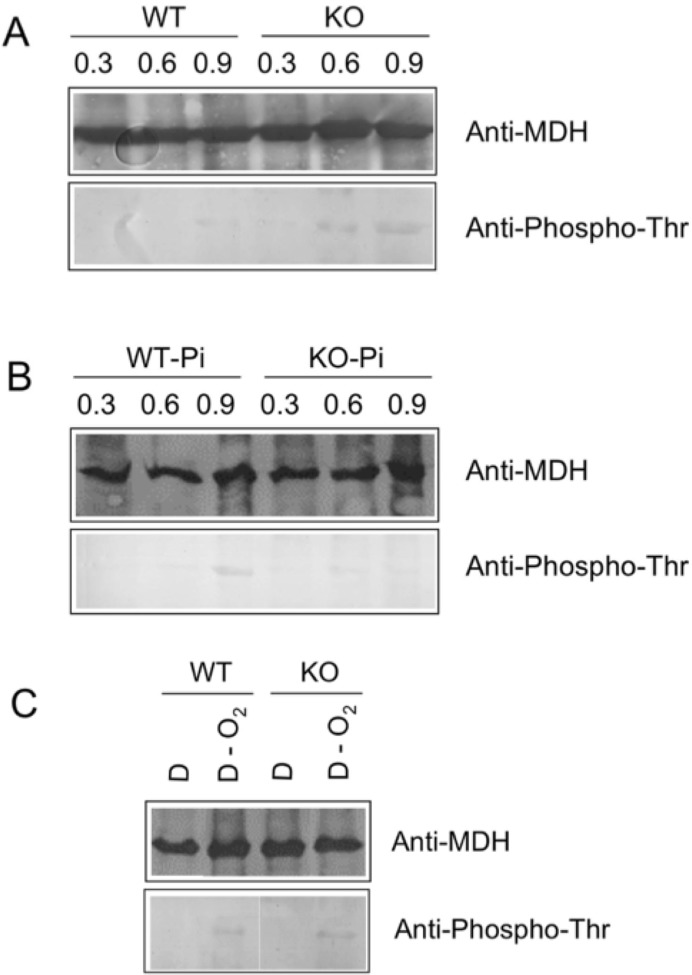
Phosphorylation of the *Mtb* MDH by *Mtb* STPKs during various bacterial growth conditions. The immunoprecipitated MDH was separated on SDS-PAGE and transferred onto nitrocellulose membranes for Western blot analyses using antibodies recognizing the MDH (anti-MDH) or phosphothreonine residue (anti-pThr). A: Bacteria cultured until early, middle and late exponential phases, (corresponding to OD_600nm_ 0.3, 0.6 or 0.9, respectively) were further cultured for 3 hours in phosphate-repleted medium or bacteria cultured until late exponential phase (corresponding to OD_600nm_ 0.9) were further cultured in phosphate limited medium. B: Bacteria cultured until OD_600nm_ 0.6 in Dubos normal medium (DM) or under deprivation of oxygen (DM-O_2_).

In order to determine whether the MDH was phosphorylated by the PknD within the bacteria, we analyzed by immunoprecipitation the MDH phosphorylation in the *Mtb* H37Rv wild-type (WT) and in the *Mtb* PknD-deficient (KO) mutant strains [30]. In normal culture condition, the MDH was always (early, middle and late exponential phases) expressed by both strains. Phosphorylated MDH was detected in the WT strain and in the PknD-KO mutant strain in middle and late exponential phases (Fig 8A). In agreement with *in vitro* experiments (Fig 4) this suggested that, *in vivo*, in normal conditions, the MDH could be phosphorylated by kinase(s) other than PknD. At the opposite, in phosphate deficient culture, the phosphorylation of MDH could be detected at the late exponential phase in the WT strain but not in the PknD-KO mutant (Fig 8A) suggesting that PknD had a more specific role in phosphorylating MDH than other kinases. When WT and PknD-KO *Mtb* were cultured in Dubos medium without oxygen, the MDH phosphorylation was detected in both strains at the middle exponential phase (OD_600_ = 0.6) (Fig 8B), implying that in hypoxia, the phosphorylation could be carried out by other STPK(s).

## Discussion

In preliminary experiments of this work we observed that PknD phosphorylated numerous proteins in *M*. *bovis*. Five of them were identified by N-terminal amino acids sequencing. In an extensive analysis of the phosphoproteome of mycobacteria, Prisic et al. (2010) reported that malate dehydrogenase (Rv 1240), an enzyme contributing to the TCA and the glyoxylate cycles and the mycobacterial integration host factor MihF (Rv 1388), the third most abundant protein of mycobacteria [39], were phosphorylated by STPKs [14]. The 3 other substrates of PknD, the iron factored superoxide dismutase A (Rv3846) essential for the survival of bacteria after intracellular infection [40], a classical nitroreductase (Rv3368C) and the alpha subunit of an electron transfer flavoprotein (Rv3028C), a protein overexpressed by INH-resistant strains and in response to osmotic stress [41], had not yet been reported as substrates of the STPKs. Our results further confirm the major contribution of these kinases, in particular PknD, as sensors/regulators of mycobacteria.

Among the five identified proteins, MDH was apparently the best substrate of PknD and it was phosphorylated both *in vitro* and in bacterial culture. Interestingly, Prisic et al. [14] identified more than 500 phosphorylation events occurring on 301 proteins in mycobacteria. Their data not only showed the MDH was phosphorylated by STPKs but mass spectrometry analysis also revealed that 3 residues could be phosphorylated. However, they did not identify whether serine or threonine were the phosphorylated amino acids. PknD is not only a STPK but also a tyrosine kinase [42] and we established that the phosphorylation of MDH by PknD occurred on threonine residues. We also showed that PknD phosphorylated the dimeric form of the enzyme. *In vitro*, 5 other STPKs (PknA, PknE, PknG, PknH and PknJ) phosphorylated MDH and four other STPKs (PknB, PknF, PknK and PknL) were ineffective. These results were confirmed by experiments using PknD-KO mycobacteria. The phosphorylation of the MDH was increased when bacteria (both WT and PknD-KO strains) reached the growth stationary phase confirming that kinases other than PknD could phosphorylate MDH. These results are reminiscent of results reported in 2006 by Perez et al. [15]. Comparing the proteins phosphorylated in WT strain and in the KOD5 strain, a PknD mutant strain, they observed that the phosphorylation of several proteins with a MW around 37 KDa and different isoelectric pH, was weaker in the KOD5 strain than in the WT strain. This is consistent with the phosphorylation of a 37 kDa protein (MDH?) mostly by PknD but also by other kinases on several phosphorylation sites. Hypoxic conditions did not affect the phosphorylation of MDH in the presence or in the absence of PknD. In phosphate-poor medium, the phosphorylation of MDH was detected in WT mycobacteria but not in the PknD-KO strain. The phosphorylation by the PknD could constitute a key step under phosphate-poor conditions, further emphasizing the role of this kinase as a sensor protein in phosphate signaling [43–46].

The phosphorylation of MDH had 2 major consequences for the enzyme. First it inhibited its *in vitro* activity. Considering that the assays were performed at saturating concentrations of substrates, the phosphorylation of the enzyme probably affected the maximal velocity of the enzyme rather than its affinity for its substrates. Second it also promoted its interaction with other proteins. Rv0020c and Rv1827, two of the five *Mtb* FHA-containing proteins. This suggested that the interactions between these proteins were phosphothreonine-dependent, in agreement with the FHA domain characteristics. The Rv1827/GarA which is regulated by phosphorylation via STPK [28], has also a phospho-independent domain capable of interacting with three non-phosphorylated metabolic enzymes (α-ketoglutarate decarboxylase, NAD^+^-dependent glutamate dehydrogenase and the α-subunit of glutamate synthetase) inhibiting their contribution to the TCA cycle and to the synthesis of glutamate [27, 47]. The interaction of MDH with the Rv0020c and the Rv1827/GarA proteins might participate to the regulation of the intermediary metabolism by FHA-containing proteins. It should be noted that the phosphorylation of MDH inhibited its activity *in vitro*, in the absence of FHA-containing proteins, demonstrating that the interaction between these proteins and MDH was not involved in the short-term regulation of MDH by phosphorylation. This interaction might play an intermediate role in targeting the phosphorylated MDH to proteolysis [48].

Within a granuloma, mycobacteria are exposed to hypoxic, acidic conditions and also to low concentrations of nutrients and high concentrations of reactive oxygen and nitrogen intermediates. In phagolysosomes mycobacteria are also exposed to a phosphate-depleted medium [49]. The phosphorylation of the MDH by STPKs when *Mtb* entered the stationary phase, when *Mtb* suffered from lack of phosphate or when *Mtb* grew under oxygen deprivation and the subsequent inhibition of the catalytic activity of the enzyme, suggest that this regulation might be one of the mechanisms of environmental adaptation allowing *Mtb* to survive under poor nutrients and/or hypoxic conditions and entering into a nonreplicating or dormant form [32, 50]. Our results confirm that PknD contributes to this regulation. The extracellular C-terminal domain of this kinase is a sensor domain which forms a funnel-shaped propeller structure containing six blades arranged cyclically around a central pore [51]. A flexible domain connects this extracellular domain to the transmembrane domains and after activation, mediates signaling through activation of the intracellular domain with the kinase activity. Recent data by Baer et al. showed that PknD was cross-phosphorylated by PknB and by a cascade successively involving PknH and PknE [52]. Considering that different signaling pathways converge to PknD and that this kinase autophosphorylates and autocatalytically increases its activity [53], PknD might thus be a major step in the regulation of the metabolic activity of the mycobacteria in the granuloma.
